# Myelitis in a Dog; Recovery

**Published:** 1896-05

**Authors:** Robert W. Ellis

**Affiliations:** New York City


### MYELITIS IN A DOG; RECOVERY.

BY ROBERT W. ELLIS, D.V.S.,
NEW YORK CITY.

ON January 9th last, I found the subject, a fox-terrier dog, about eight years of age, suffering with paraplegia of hind extremities involving the rectum and bladder. There was temporary anorexia, which I attributed to the fact that constipation had existed for two or three days. Although he could not stand or even wag his short butt of a tail, he would respond to his name when called, by travelling after you on his forelegs, trailing his hind ones after him, the muscles of which were so relaxed that the limbs would cross each other when he would turn from a straight course.

I began treatment by administering a dose of "salts," which I have found to be quite as responsive in the canine as in the human subject, and very convenient, as it can be carried in the pocket, so that you can dissolve a teaspoonful and administer it at a moment's notice, if requisite. I have adopted its use almost entirely in canine practice to the exclusion of the oleaginous form of purge. I immediately followed the administration of the purge by the application of electricity to the lumbar region and hind extremities, and during its application there was a decided movement of the bowels and voiding of urine. At the first application of the electric current, and at the next three or four, the dog seemed to suffer no pain, but after about the fifth or sixth application there seemed to be hyperæsthesia of the parts, they being very sensitive to the touch, and a look of dread seemed to pass over the dog's countenance at the sight of the battery; also there was a continual complaining during its application. The next morning after the first use of the battery and movement of the bowels the appetite had returned and remained good from that time on. At this time I put the patient on tr. nucis vomicæ, ♏iij, three times a day, which I steadily increased until it reached ♏xx three times a day. I continued the use of the battery twice a day, and at each applition of it, much to my discomfort, although undoubtedly for the patient's good, defecation and micturition took place.

Recovery was gradual, so that at the end of about two weeks he was able to bear his weight on the limbs if they were placed in a standing position and a hand was placed against each hip to prevent him falling sidewise, and at the end of three weeks he was able to get up himself and stand, and a day or two later ventured, on being called, and found he could walk, although if he attempted to run he would fall.

At this point I began to decrease the doses of nux vomica and to use the battery only once a day and then every second day, and finally, about ten days from the time of his beginning to walk, I ceased its use altogether, also that of the nux vomica. After he began to walk his bowels and bladder resumed their normal functions, and I was no longer annoyed at the time of using the battery. The dog at the present time can walk or run at will, has an enormous appetite, and has grown very stout and heavy.

There is still noticeable at times a lack of *entire* control of the hind extremities, or when turning a corner short in chasing a cat; but I am in hopes that will entirely disappear with the return of the dry weather of the summer season.

